# Decompressive craniectomy for malignant middle cerebral artery infarction in a 16-year old boy: a case report

**DOI:** 10.1186/s13256-016-1145-7

**Published:** 2016-12-20

**Authors:** Simon Lammy, Paul Fivey, Meharpal Sangra

**Affiliations:** Neurological Surgery (Neurosurgery), Department of Neurosurgery, Institute of Neurological Sciences, 1345 Govan Road, Glasgow, G51 4TF UK

**Keywords:** Cryptogenic, Decompressive, Craniectomy, Vasospasm, Stroke

## Abstract

**Background:**

Cryptogenic stroke frequently occurs in younger patients and has a high risk of recurrence. Consequently, secondary prevention is often suboptimal as there is no known risk factor to target. This case demonstrates an unexpected finding of middle cerebral artery infarction and extensive malignant transformation in a 16-year-old boy more than a day post-admission. The lack of a proven culprit lesion makes this case even more intriguing and subsequently raises questions of cryptogenic mechanisms in the context of unrelated trauma.

**Case presentation:**

A 16-year-old white boy had been stabbed in his chest but had a Glasgow Coma Scale score of 15. Over a day later he developed sudden signs and symptoms consistent with a neurological event of unknown etiology. Computed tomography demonstrated significant cerebral edema but was equivocal in its list of differentials. A computed tomography scan of his chest demonstrated no cardiac wall or vascular injury and he was transferred to our neurosurgical unit for intracranial pressure monitoring. A computed tomography angiogram revealed an unexpected finding of malignant middle cerebral artery infarction. Failure to medically manage his intracranial pressure resulted in a decompressive craniectomy less than 12-hours postictus. Despite extensive diagnostic investigations no culprit lesion was identified and no patent foramen ovale found. Since discharge he has returned to full functional status. He was the youngest patient (mean age of 43 years) out of a 10-year institutional retrospective on decompressive craniectomies for malignant middle cerebral artery infarction (*n* = 40) and had the singularly best Glasgow Outcome Scale score of 5.

**Conclusions:**

This case highlights the preponderance of cryptogenic stroke in younger patients and its etiological elusiveness. It further demonstrates that age is predictive in terms of survival and functional outcome in the context of malignant middle cerebral artery infarction.

## Background

In the UK, stroke is the fourth leading cause of death and the largest cause of complex disability [1, 2]. The incidence of malignant middle cerebral artery infarction (mMCAI) is 1% of all strokes (approximately 1520 patients per annum) [2] and carries an 80% 30-day mortality if neurosurgical intervention does not occur [1]. With infarction of greater than 50% of middle cerebral artery (MCA) territory, a significant mass effect and concomitant herniation of brain tissue may occur resulting in rapid demise. Recent randomized controlled trials (RCTs) and subsequent meta-analysis make it clear that surgical intervention and subsequent survival and functional outcome are inversely proportional to age [1]. Furthermore, cryptogenic stroke frequently occurs in younger patients and due to it being notoriously difficult to identify a culprit lesion secondary prevention is often suboptimal [1, 2].

## Case presentation

A 16-year-old white boy presented due to being stabbed in his chest. He had a blood alcohol level of 145 mg/dL and was hemodynamically compromised but had no head trauma: his Glasgow Coma Scale (GCS) score was 15. A plain chest X-ray (CXR) confirmed a left-sided pneumothorax. Insertion of a 32 Fr chest drain expelled 100 ml of fresh blood. He was admitted under our general surgical team and was making a good recovery.

On the second post-admission day he experienced sudden onset left leg hemiplegia: Medical Research Council (MRC) Grade 1. He was Babinski positive and had hemineglect. He became incontinent of urine, his GCS dropped to 11 (E3 V3 M4), and his pupils were reactive at size 4. An electrocardiogram (ECG) showed ST-segment elevation but his troponin I was not elevated.

**Fig. 1:**
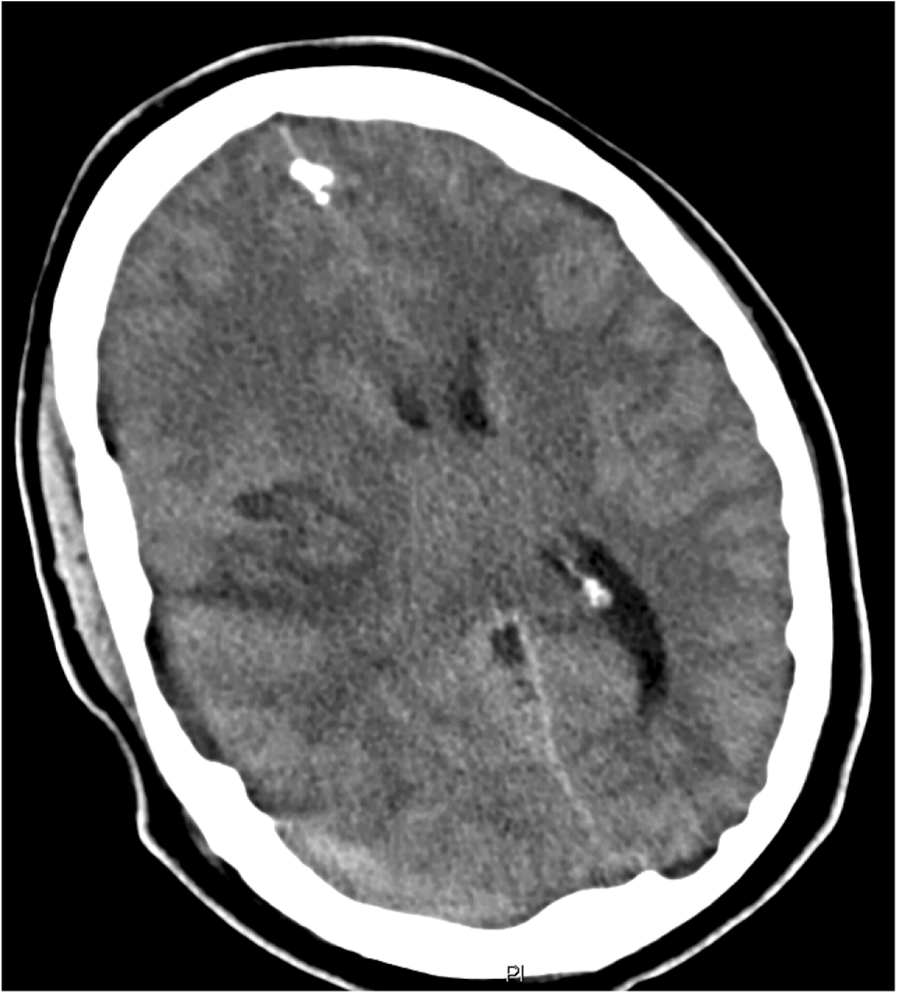
Computed tomography (axial) demonstrating a right-sided lesion of indeterminate nature causing mass effect and significant midline shift

A computed tomography (CT) scan of his head at 0918 hours (Fig. 1) demonstrated a right-sided 8 × 7 × 7 cm mass causing left ventricular dilation, 10 mm midline shift, right hemispheric sulcal effacement, and early uncal herniation. This was unexpected due to absence of vessel hyperdensity and no head injury.

A CT scan of his chest demonstrated a potential non-penetrating pericardial injury and pericardial and pleural effusions but no obvious ventricular wall injury. A transthoracic echocardiogram (TTE) revealed no ventricular wall motion derangement. He was intubated for transfer to our neurosurgical intensive care unit (ICU) that morning.

On admission to our neurosurgical ICU he had no eye movements but was localizing to pain. He was not coagulopathic and his mean arterial blood pressure (MAP) was 95 mmHg, an intracranial pressure (ICP) bolt inserted in the ICU recorded an ICP of 38 mmHg, and his cerebral perfusion pressure (CPP) was 57 mmHg.

**Fig. 2:**
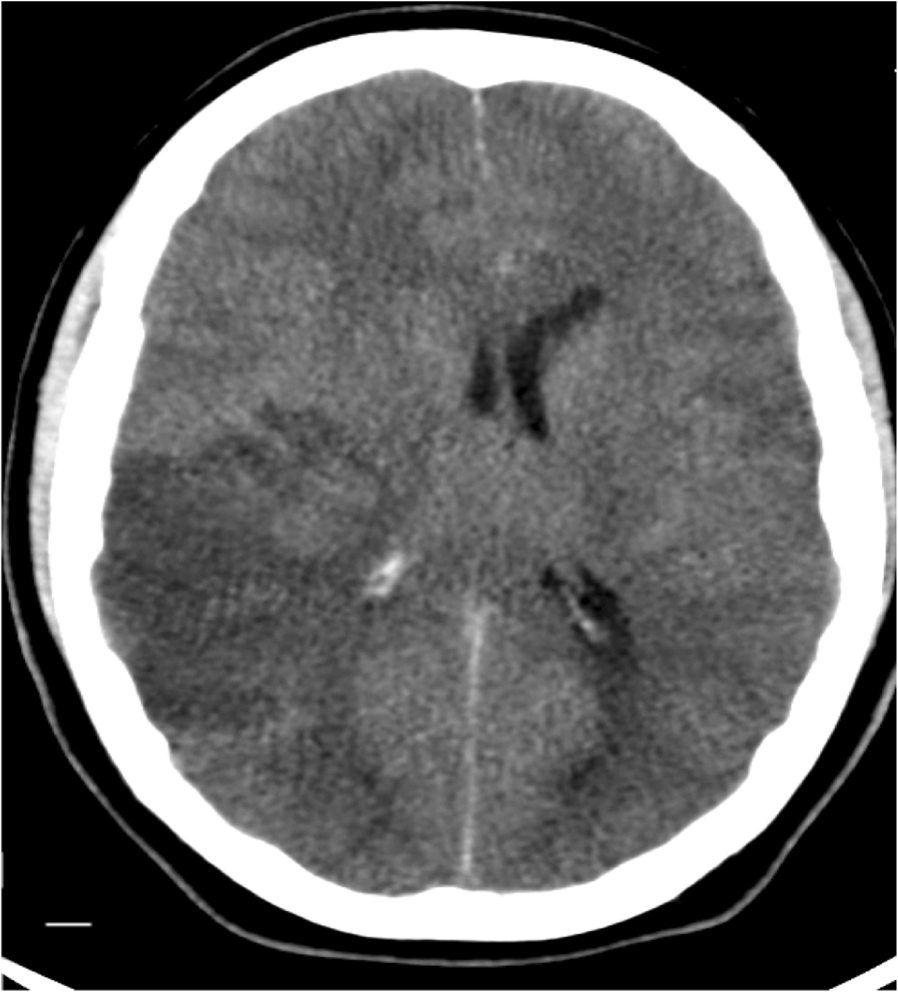
Computed tomography (axial) demonstrating a large middle cerebral artery infarct and concomitant perilesional edema

**Fig. 3:**
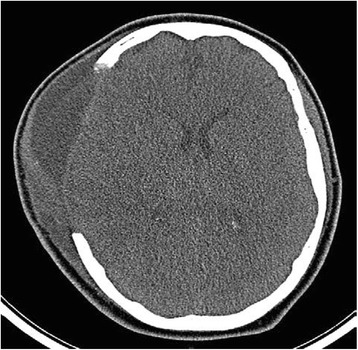
Computed tomography (axial) showing a large right-sided craniectomy and expansion of brain parenchyma away from the midline

A repeat CT scan of his head at 1645 hours (Fig. 2) demonstrated a 6 mm midline shift, complete obliteration of his basal cisterns, and generalized sulcal effacement (100 ml 20% mannitol was given). A thoracic CT angiogram was conducted to exclude occult pathology but this was normal. An unexpected finding on his cranial CT angiogram was mMCAI. It was decided that an aggressive surgical approach was warranted, that is, a right-sided decompressive craniectomy, and this was immediately carried out (Fig. 3). The ICP bolt was removed intraoperatively.

On the second postoperative day he was localizing to pain but remained hemiplegic. A repeat TTE demonstrated good systolic function and a small pericardial effusion but no tamponade, thrombus, or septal defects. He was extubated on the fifth postoperative day and discharged from ICU.

He made excellent progress over a month and had no focal neurological deficits. Aspirin at 150 mg daily was commenced but not warfarin (as no thrombus was proven). A laboratory examination revealed no lupus anticoagulant and cardiolipin antibodies, no family history of clotting disorders, and a normal cerebrospinal fluid (CSF) protein. A repeat TTE demonstrated no regional wall abnormality (and no septal defects), a bubble test at 4-weeks postictus was normal, and CT angiograms of his aorta and aortic arch were once again both normal. No magnetic resonance imaging (MRI) was conducted based on advice from the stroke physicians because the embolic lesion was clearly visible on CT. At 3-months postictus he was fully independent having a Glasgow Outcome Scale (GOS) score of 5. Three titanium cranioplasties were required over a 3-year period for infective complications. He now drives a car and is in full-time employment.

## Discussion

Cryptogenic stroke accounts for 23 to 45% of all strokes and frequently occurs in younger patients [1–5]. It has a high risk of recurrence and subsequent incidence of transient ischemic attacks (TIAs). As no clear underlying etiology exists adherence to treatment is often suboptimal (as there is nothing to target). The use of advanced diagnostic techniques includes: long-term monitoring to capture paroxysmal atrial fibrillation (AF); high-resolution MRI to detect plaque, dissection, or vasculitis; and CT angiography to evaluate aortocardiac embolism to identify culprit lesions to target [3, 4].

Due to the cause remaining elusive, his cryptogenic stroke was probably transitory being triggered by his original trauma. This is in keeping with cryptogenic stroke as diagnostic modalities may remain normal even if delayed until after the acute illness has passed [3, 4]. Our patient did not undergo 24-hour Holter monitoring as AF detection rates using this modality are inferior to serial ECGs (these did not reveal AF) and implantable long-term cardiac monitoring was not indicated [4, 5].

Further imaging using TTE and CT angiography excluded cardiac lesions, paradoxical and aortogenic embolism, atrial septal aneurysm, aortic atheroma, and arterial dissection. A patent foramen ovale (PFO) was ruled out on multiple TTEs [4, 5].

Other transitory causes include vasospasm, hypercoagulable states, for example sickle cell disease [3, 4], and migraine-induced stroke [3, 4]. All, except vasospasm, were excluded as he did not undergo digital subtraction angiography (DSA). Consequently, it might remain cryptogenic as TTE is considered the gold standard investigation in the evaluation of cryptogenic stroke (and he had three).

A coagulopathy was excluded and three TTEs were undertaken: the use of transesophageal echocardiography (TEE) is equivocal [4–7]. Although clinical (for example diabetic), genetic (for example clotting disorders), and electrophysiological (for example premature atrial complexes, left atrial dilatation, and reduced left ventricular ejection fraction) causes were excluded some hematological biomarkers, for example pro-brain natriuretic peptide, were not investigated [7]. According to recent research, these might be predictive for incident AF in cryptogenic stroke [4]. Karaaslan *et al.* [8] demonstrated that an etiology can challengingly remain elusive.

Karaaslan *et al.* [8] described a case of cryptogenic stroke in a 19-year-old woman undergoing elective laparotomy for resection of a pancreatic pseudopapillary tumor. There had been significant intraoperative blood loss requiring an ICU admission postoperatively. Once extubated 12 hours later she was unable to speak and had a left facial droop. A CT scan of her head demonstrated bilateral thalamic infarcts (subsequently confirmed on MRI) and treatment included anticoagulation. Subsequent investigations including TTEs, TEEs, and genetic, collagen, vascular, and coagulation profile studies failed to identify a potential source of embolic stroke, for example a PFO did not exist, and failed to demonstrate any physiological abnormalities that might have generated a hypercoagulable or coagulable state. She made a full recovery except for minimal memory deficits [7, 8].

Potential sources of our patient’s culprit lesion include a paradoxical embolism because a probe-patent PFO may allow right to left shunting only under specific conditions and toxins as he was found in an isolated street.

## Conclusions

This case demonstrates a fact that mMCAI can present in a myriad of ways and is potentially devastating. Although he made a full recovery he remains at high risk of future strokes due to the cryptogenic nature of his original presentation despite being thoroughly investigated (including genetic studies) [3, 5]. Clearly, more research is needed into this particular type of stroke to identify a potential source for culprit lesions as cryptogenic stroke has a predilection for otherwise young healthy patients rendering them to a lifetime of future strokes and serious morbidity.

## Abbreviations

AF: Atrial fibrillation
CPP: Cerebral perfusion pressure
CSF: Cerebrospinal fluid
CT: Computed tomography
CXR: Chest X-ray
DSA: Digital subtraction angiography
ECG: Electrocardiogram
GCS: Glasgow Coma Scale
GOS: Glasgow Outcome Scale
ICP: Intracranial pressure
ICU: Intensive care unit
MAP: Mean arterial blood pressure
MCA: Middle cerebral artery
mMCAI: Malignant middle cerebral artery infarction
MRC: Medical Research Council
MRI: Magnetic resonance imaging
PFO: Patent foramen ovale
RCTs: Randomized controlled trials
TEE: Transesophageal echocardiography
TIA: Transient ischemic attack
TTE: Transthoracic echocardiogram
